# Perceived Public Stress Among Jordanians During the COVID-19 Outbreak

**DOI:** 10.1017/dmp.2020.328

**Published:** 2020-09-09

**Authors:** Mariam H. Abdel Jalil, Mervat M. Alsous, Eman A. Hammad, Rimal Mousa, Mais M. Saleh, Khawla Abu Hammour

**Affiliations:** Department of Biopharmaceutics and Clinical Pharmacy, School of Pharmacy, University of Jordan, Amman, Jordan; Department of Pharmacy Practice, Faculty of Pharmacy, Yarmouk University, Irbid, Jordan; Department of Pharmaceutics and Pharmaceutical technology, School of Pharmacy, The University of Jordan, Amman, Jordan

**Keywords:** COVID-19, Jordan, PSS-10, stress, students

## Abstract

**Objectives:**

Outbreaks and containment measures implemented to control them can increase stress in affected populations. The impact of the coronavirus disease 2019 (COVID-19) outbreak on perceived stress levels in the Jordanian population is unknown. The aim of the study was to determine the perceived stress level and factors associated with it in the Jordanian population during the COVID-19 outbreak.

**Methods:**

Required data, such as those from the Perceived Stress Scale (PSS-10) and possible predictors of perceived stress, were collected through a Web-based survey. Statistical analysis was conducted through SPSS.

**Results:**

The mean (SD) of perceived stress score was 19.8 (6.7). Regression analysis revealed that stress was increased in females, young adults, usually being stressed more than others by a health problem, increased perceived severity of the disease, increased overall worry score, and student’s worry regarding their studies/graduation. Perceived stress was decreased if participants’ self-rated health status score increased.

**Conclusions:**

In the context of increasing public health preparedness, the results of this study can be used in designing interventions to alleviate stress in susceptible segments of the Jordanian community.

The 2019 coronavirus (COVID-19) is a pandemic infectious disease that was first detected in Wuhan city, China, on December 31, 2020, and was declared as a global health emergency on January 30, 2020, by the World Health Organization.^1^ The virus spread globally at a quick pace; within less than 2 mo the number of new confirmed cases beyond the borders of China outpaced those inside the country.

Jordan is an Arabic country located in the Middle Eastern region with a population estimated of 10.20 million. On March 2, the first case of coronavirus was confirmed. On March 17, the Jordanian government declared a state of emergency to limit the spread of the virus. It also implemented a strict curfew; public gathering was banned; schools and universities were closed and learning was switched to online; when possible, work was switched also to online for public and private employees.

Expectedly, people isolated at home will experience boredom and frustration due to loss of usual routine and reduced social activity. Moreover, financial loss due to the interruption of work will impose a financial pressure on people.^2^ People are also likely to experience fear of being infected or their loved ones being infected, which could generate feelings of stress, helplessness, and stigma.^2^

The present study aimed to assess the perceived stress during the COVID-19 outbreak and to underline sources of stress in the Jordanian community.

## METHODS

### Ethical Approval

The protocol of the study was approved by the Institutional Review Board of Jordan University hospital (decision no. 2020/74).

### Target Population and Data Collection Tool

The targeted population was the general public of Jordan aged 18 y or older. The sampling technique attempted to scope a wide spectrum of participants through convenient and snow ball sampling. The survey was anonymous, and it was distributed using an electronic format, through Google Forms. The link to the survey, in addition to an introductory page about the survey, were sent by means of social media platforms, such as Facebook, Messenger, Twitter, and Whatsapp. The survey instrument was initially developed by the authors by means of an extensive review of the available literature.^3-6^ The questionnaire was in Arabic, it was reviewed for content validity by 3 PhD holders with experience in conducting such research, it was also subjected to pilot testing using a small group of participants (*n* = 13) from the general public with various backgrounds (working in the medical field or other fields) and ages (older than 35 and younger than 35). They reviewed the questionnaire and suggested modification to the original questionnaire based on the developments of the outbreak in Jordan. For instance, 1 of the important additions that was made based on the pilot study was the addition of a question regarding the students’ worry about their studies/graduation that could be caused by lockdown and switching teaching to online.

The final version of the survey was composed of 6 sections. The first section aimed to collect general information about the participants, such as age, gender, and a single item self-rated health status “how do you rate your general health”^7^ on a scale of 1-10, with 1 indicating poor health and 10 indicating excellent health. The second section contained general questions designed to evaluate the participants’ knowledge regarding COVID-19 disease in terms of general facts, transmission modes, presence of treatments, and common misinformation, the knowledge scale ranged from 0 to 13, it was adapted from a previous study of ours, and was modified to suit the general public.^8^ The third section consisted of 9 questions about recent changes in behaviors since the beginning of the outbreak in Jordan, such as changes in hygienic behaviors, including hand hygiene, social avoidance, and avoidance of specific places, and personal protective behaviors, such as wearing masks. Behaviors were grouped into 3 categories; participants reporting that they increased the frequency of performing protective behaviors compared with usual since the outbreak, no change in behaviors, and a change toward nonprotective behaviors (ie, performing protective behaviors less frequently compared with usual since the outbreak spread in Jordan), the questions were selected based on previous research^5^ and expected local response to the virus. The fourth section evaluated the types of information sources for COVID-19 related knowledge, how much each source was trusted, and the time spent hearing COVID-19 related news. It also evaluated, through 5 questions, the perceived sufficiency of information received about various aspects of the disease, the overall scale for these questions was from 0 to 5. The fifth section was related to general questions to assess the perceptions of the public regarding the perceived severity of the disease on a scale of 1-10, the controllability of the outbreak, perceived self-efficiency in preventing the disease^9^ (0-3 scale), and the necessity of lockdown. Moreover, it also included 2 questions regarding perceived susceptibility to getting COVID-19 disease,^4^ those scales were summed on a scale from 0 to 6. Another part of this section was related worries associated with COVID-19 that the Jordanian population experiences, these include worries regarding financial income, ability to secure food; medicine; and worrying that COVID-19 might infect the individual or a family member. These questions were grouped and scored on a scale from 0 to 10. Separately, participants were asked if they are worried about their studies due to the COVID-19 disease and lockdown. The last section was the Perceived Stress Scale-10 (PSS-10).^6^ PSS-10 is a validated tool that is used to measure perceived stress levels over the past month, and has been used to measure stress in various populations, including elderly populations, pregnant women, in addition to health-care workers and students.^10^ The total score of the tool range from 0 to 40, with higher scores indicating higher perceived stress.

### Sample Size Calculation and Statistical Analysis

The minimum required sample size was calculated using the online Raosoft sample size calculator (http://www.raosoft.com/samplesize.html?nosurvey) and was 385 participants. Collected data were processed using SPPSS version 22. A chi-squared test was used to detect differences between categorical data. To determine the relationship between PSS-10 scores and the independent categorical variables, a t-test was used for binomial variables, while 1-way analysis of variance (ANOVA) was used for multinomial variables. For continuous variables, the Pearson correlation coefficient was computed. Variables that were statistically associated with PSS-10 scores were included in the multiple linear regression analysis. Statistical significance was considered at *P* < 0.05. To exclude careless responses, we used a dichotomous self-reported single item indicator.^8,11^

## RESULTS

From March 24, 2020, until April 7, 2020, we received a total of 1104 responses; of those, 71 were excluded from the analysis (15 declined to take part, 26 reported careless responses, 30 were less than 18 y). Thus, we totally analyzed 1033 responses. Amongst those, 56 reported being physicians, 33 reported being nurses, 39 reported being pharmacists; those will be referred to as health-care providers (HCP) throughout the text. General characteristics of the included participants are presented in Table 1. The daily number of newly detected cases in Jordan during the study period ranged between 4 cases per day and 40 cases per day (https://www.worldometers.info/coronavirus/country/jordan/).

**TABLE 1:** General Characteristics of the Study Population (*n* = 1033)

Characteristic	n (%)	Mean Stress Level (SD)^**a**^
Age		
18-25	355 (34.4%)	21.6 (7)
26-35	326 (31.5%)	20.2 (6.2)
36-45	166 (16.1%)	18.6 (6.2)
46-55	125 (12.1%)	17.7 (5.8)
>55	61 (5.9%)	15.0 (7.2)
Gender		
Male	349 (33.8%)	17.7 (6.5)
Female	684 (66.2%)	20.9 (6.6)
Monthly income (JOD)		
<250	88 (8.5%)	21.4 (6.1)
250-499	203 (19.7%)	19.8 (7.2)
500-749	212 (20.5%)	20.2 (6.8)
750-999	173 (16.7%)	19.5 (6.9)
1000-1499	167 (16.2%)	19.8 (5.8)
1500-1999	75 (7.3%)	19.1 (7.5)
≥2000	115 (11.1%)	18.9 (6.3)
Smoker		
Yes	311 (30.1%)	19.8 (6.3)
No	670 (64.9%)	19.8 (6.9)
Ex-smoker	52 (5.0%)	19.2 (6.4)
General health perception (on a scale of 10)	8.4 (1.3)^a^	
Marital status		
Single	501 (48.5%)	21.1 (6.8)
Married	504 (48.8%)	18.6 (6.4)
Other	28 (2.7%)	19.7 (6.8)
Pregnant?		
Yes	26 (3.8%)	20.9 (6.6)
No	658 (96.2%)	20.2 (6.6)
Have elderly of first-degree relatives?		
Yes	659 (63.8%)	19.8 (6.6)
No	374 (36.2%)	19.8 (6.9)
Are you or any of your friends or relatives infected with COVID-19?		
Yes	28 (2.7%)	20.3 (5.6)
No	1005 (97.3%)	19.8 (6.7)
Perceived susceptibility scale	2.9 (1.4)^a^	
Overall stress level	19.8 (6.7)^a^	

a Expressed as mean (SD).

### Participants’ Knowledge and Sources of Information

The mean (SD) of the knowledge scale was 10.3 (1.7) (out of 13) in the entire study population; there were no differences in knowledge score based on age (younger or older than 35 y) or gender. The mean knowledge score in HCP 10.8 (1.4) was slightly but significantly higher than the non-HCP participants 10.2 (1.7). The main sources of information reported by participants are shown in Supplemental Figure 1. The most trusted source of information was global websites, which were completely trusted by 71.6% of respondents. In terms of sufficiency of information, more than 92% of respondents reported that they received sufficient information regarding symptoms of the disease, preventive measures, and numbers of infected cases or COVID-19 fatalities in Jordan. However, only 48.7% reported receiving sufficient information regarding COVID-19 treatments. HCP had greater perceived sufficiency of information (66.4%) regarding COVID-19 treatment compared with the rest of the population (46.3%).

### Trust in Governmental Actions

As many as 59.7% of respondents expressed great trust in the ability of the MOH to handle the outbreak. Furthermore, as much as 94.9% agreed that lockdown was necessary to prevent the spread of the virus.

### Behavioral Responses to COVID-19

As shown in Supplemental Table 1, the majority of participants (>90%) adapted protective behaviors, such as washing hands and avoiding handshakes, while a smaller proportion of participants increased their consumption of citrus fruits and vitamins (59.1%), or the frequency of wearing masks (59.2%).

### Stress Level During the COVID-19 Outbreak

Overall mean (SD) of stress score was 19.8 (6.7). Results of single variable analysis are portrayed in Supplemental Table 2; of the 18 tested predictors that were tested, 10 were found significant. During multivariate linear regression analysis, 7 of those predictors were significant. The results of multivariate regression analysis are shown in Table 2. The coefficient of determination (R^2^) for the current model was 0.271.

**TABLE 2:** Multiple Linear Regression Analysis Results

Variable	Unstandardized B	SE	95% CI	*P*-Value
Female gender	2.3	0.39	1.8 – 3.0	**<0.001**
Younger than 36 years	2.3	0.42	1.4 – 3.2	**<0.001**
Perceived health status scale	−0.4	0.15	−0.69 – −0.11	**0.007**
Usually being stressed more than others by a health problem	2.8	0.58	1.6 – 3.9	**<0.001**
Hearing news more than 6 hours per day	0.98	0.58	−0.14 – 2.12	0.09
Trust MOH	−0.79	0.38	−1.5 – 0.062	0.03
Perceived susceptibility scale	0.18	0.13	−0.08 – 0.44	0.18
Perceived severity of the disease	0.34	0.11	0.12 – 0.56	**0.002**
Worry scale (0-10)	0.78	0.08	0.63 – 0.93	**<0.001**
I am worried that the COVID-19 will affect my studies/graduation	2.02	0.42	1.2 – 2.8	**<0.001**

### COVID-19 Associated Worries

A total of 32.7% of the participants reported that they were worried about the impact of the disease on their studies/graduation. As much as 30.5% of the Jordanians were highly worried that the disease may affect a family member, while 18.7% of respondents were greatly worried that the disease might affect them personally. Furthermore, respondents were greatly worried that the disease will impact their income (23.9%) and ability to obtain their food (22.2%) and drugs (18.7%).

### Limitations

The use of online platforms is inherently associated with certain limitations that include the availability of Internet coverage and the technological knowledge required to take part in such surveys. This could explain the reason for the low percentage of participants older than 55-y-old in the present survey. Although the PSS-10 scale was validated, other scales used in this study, such as worry scale, were not. In addition to literature review, they were also developed in response to the developments of the COVID-19 outbreak in the Jordanian community. Furthermore, our sample was predominantly female (66.2%). This high proportion of participating females could indicate a greater interest of women in taking part in such studies, such a trend was also reported by other researchers.^1^

## DISCUSSION

The present study was conducted during the COVID-19 outbreak in Jordan. The mean stress score was higher than the score observed in study of Limcaoco et al.^1^ (17.4 [6.4]), a study that was conducted through an anonymous on-line survey distributed by means of social media in 41 countries during the period of the 17^th^ of March to the 1^st^ of April, 2020. The higher stress score noted in females is not an unusual finding, since it has been reported in the context of COVID-19 and other stressful conditions.^1,12^ There was a decreasing trend in mean stress as age increased. Despite being unexpected, a similar trend was reported during the COVID-19 outbreak by Limcaoco et al.^1^ During SARS in Hong Kong, higher job-related stress was noted in the younger (≤33 y) frontline health-care workers.^13^ Our regression analysis results showed that being a young adult (18-35 y) was a predictor of increased perceived stress compared with ≥ 36 y. This is an interesting finding; it is estimated that the population of Jordan aged between 18 and 35 accounts for more than a quarter of the Jordanian population. Potentially these are parents, university students, and employees at various jobs. Social support programs should be targeting the most vulnerable individuals. Further studies are recommended to understand underlying reasons of stress and create strategies to appropriately deliver support to the subgroups of population that most need it. Of note, 32.7% of respondents were worried about possible influence on their studies, which could be related to switching from traditional teaching to online teaching. Such change can stress students due its novelty, coupled with the fact that many students did not have a suitable working environment or even appropriate tools to support such a learning approach. This highlights a key role for academics and the Jordanian Ministry of Higher Education to take appropriate measure to alleviate this stress.

The COVID-19 crisis has made people across the world economically vulnerable, due to complete or partial loss of income. Our survey was conducted during the second week of lockdown in Jordan for a period of 2 wk. During that period, approximately 23.0% of respondents expressed that they were greatly worried about their income and their ability to get food. Such worries were also reported in developed countries. In the United Kingdom for instance, a preliminary report on food insecurity (accessed through https://foodfoundation.org.uk/) reported that 21.6% of adults felt very worried or fairly worried about getting the food they need during COVID-19 crisis. Of interest, almost all surveyed Jordanians endorsed lockdown, despite its negative sequels on their ability to work. To a lesser extent, endorsement of strict preventive measures has been also reported in the United States.^3^

An encouraging result was that the MOH was trusted by Jordanians to handle the disease. Previous research in the United Kingdom during H1N1 outbreak reported that respondents reporting higher trust in the government and responding agencies were more likely to adhere to their guidance.^5^ Our study revealed that a significant proportion of respondents adopted protective behaviors, this could be due to its high transmissibility and rapid global circulation.

## CONCLUSIONS

A sample of Jordanians’ perceived stress levels were measured for the first time in Jordan, and several implicated factors were identified. Managing such stress is an important goal that needs to be achieved, especially in the absence of an effective vaccine or antiviral therapy, to build supportive plans and decrease population vulnerability to stress, as increased mortality has been associated with increased levels of stress.^14^ Improving communication between authorities and the general public is necessary, especially in issues related securing income, food, and medical supplies. Moreover, academics need to plan appropriate interventions to manage stress in students.

## Supplementary material

For supplementary material accompanying this paper visit https://doi.org/10.1017/dmp.2020.328. click here to view supplementary material
