# Supplementary material for: Perceived Public Stress Among Jordanians During the COVID-19 Outbreak
Source: Disaster Med Public Health Prep. 2020 Sep 9:1–5. doi: 10.1017/dmp.2020.328 (PMC7642498; doi:10.1017/dmp.2020.328)
Supplement: Supplementary file 1 [file S1935789320003286sup.zip › S1935789320003286sup002.docx]

Figure 1: Reported sources of COVID-19 related knowledge
