# Supplementary material for: Perceived Public Stress Among Jordanians During the COVID-19 Outbreak
Source: Disaster Med Public Health Prep. 2020 Sep 9:1–5. doi: 10.1017/dmp.2020.328 (PMC7642498; doi:10.1017/dmp.2020.328)
Supplement: Supplementary file 1 [file S1935789320003286sup.zip › S1935789320003286sup003.docx]

**Perceived public stress among Jordanians during the COVID-19 outbreak**

*** Supplementary material 3**

| **Results of single variable analysis** | | | | |
| --- | --- | --- | --- | --- |
| Categorical variables (student t-test) | | | | |
| Variable | N | Mean (SD) | T | p-value |
| **Gender**  Female  Male | 349  684 | 20.9 (6.6)  17.7 (6.5) | 7.2 | **<0.001** |
| **Age**  ≤35  >35 | 681  352 | 20.9 (6.6)  17.7 (6.3) | 7.6 | **<0.001** |
| **Being a health care provider (HCP)**  Yes  No | 128  905 | 19.3 (6.1)  19.9 (6.8) | -0.987 | 0.32 |
| **Has elderly?**  Yes  No | 659  374 | 19.8 (6.6)  19.8 (6.9) | 0.026 | 0.98 |
| **Income less than 500 JOD**  Yes  No | 291  742 | 20.3 (7.0)  19.6 (6.6) | 1.35 | 0.18 |
| **Smoker?**  Yes  No | 311  722 | 19.8 (5.7)  19.8 (6.6) | 0.016 | 0.99 |
| **Usually being stressed more than others by a health problem**  Yes  No | 122  911 | 24.4 (5.7)  19.2 (6.6) | 9.2 | **<0.001** |
| **Hearing news more than 6 hours per day**  Yes  No | 123  910 | 21.7 (7.2)  19.6 (6.6) | 3.3 | **0.001** |
| **Trust Ministry of Health**  Yes  No | 617  416 | 19.4 (6.8)  20.4 (6.5) | -2.47 | **0.013** |
| **Media overrates the corona threat**  Yes  No | 179  854 | 20.6 (6.9)  19.6 (6.7) | 1.7 | 0.08 |
| **I am worried that the COVID-19 will affect my studies/graduation**  Yes  No/not a student | 338  691 | 22.1 (6.5)  18.7 (6.6) | 0.8 | **<0.001** |
| **Continuous predictors** | | | | |
| **Perceived health status on a scale of 10:** |  |  | -0.072* | **0.02** |
| **Perceived severity of the disease** |  |  | 0.147* | **<0.001** |
| **Perceived susceptibility scale** |  |  | 0.094* | **0.003** |
| **I can prevent myself from being infected with the Coronavirus by taking appropriate preventive measures (self-efficiency)** |  |  | -0.06* | 0.052 |
| **Knowledge scale (0-13)** | - | - | -0.036* | 0.246 |
| **Worry scale (0-10)** | - | - | 0.359* | **<0.001** |
| **Newly diagnosed cases each day** | - | - | -0.022* | 0.47 |
| * Pearson correlation coefficient | | | | |
