# Supplementary material for: Perceived Public Stress Among Jordanians During the COVID-19 Outbreak
Source: Disaster Med Public Health Prep. 2020 Sep 9:1–5. doi: 10.1017/dmp.2020.328 (PMC7642498; doi:10.1017/dmp.2020.328)
Supplement: Supplementary file 1 [file S1935789320003286sup.zip › S1935789320003286sup001.docx]

**Perceived public stress among Jordanians during the COVID-19 outbreak**

*** Supplementary material 2**

**Behavioral responses to COVID-19 outbreak in Jordan**

| Behavior | % of respondents reporting a change towards protective behaviors | %of respondents reporting no change in behaviors |
| --- | --- | --- |
| Washing hands with water and soap. | *90.1%* | *9.6%* |
| Using alcoholic hand disinfectants. | 88.8% | 10.1% |
| Disinfecting surfaces in work or at home. | 85.8% | 13.4% |
| Wearing masks. | 59.2% | 30.5% |
| Increasing the consumptions of vitamins, citrus fruits, and herbal supplements. | 59.0% | 35.3% |
| Avoiding going to hospitals even if needed.1 | 81.6% | 14.5% |
| Avoiding social events. | 92.8% | 4.4% |
| Washing fruits and vegetables before storing them. | 60.2% | 39.8% |
| Avoiding handshaking. | 94.8% | 3.9% |
